# Engaging Black Churches to Address Cancer Health Disparities: Project CHURCH

**DOI:** 10.3389/fpubh.2018.00191

**Published:** 2018-07-19

**Authors:** Lorna H. McNeill, Lorraine R. Reitzel, Kamisha H. Escoto, Crystal L. Roberson, Nga Nguyen, Jennifer I. Vidrine, Larkin L. Strong, David W. Wetter

**Affiliations:** ^1^Health Disparities Research, University of Texas MD Anderson Cancer Center, Houston, TX, United States; ^2^Psychological Health and Learning Sciences, University of Houston, Houston, TX, United States; ^3^Family and Preventive Medicine, Stephenson Cancer Center, University of Oklahoma, Oklahoma, OK, United States; ^4^Huntsman Cancer Institute, University of Utah, Salt Lake City, UT, United States

**Keywords:** health disparities, cancer prevention, community engagement, black churches, community-based participatory research

## Abstract

African Americans in the United States suffer disproportionately from cancer, having the highest mortality rate of any racial/ethnic group across all cancers for the past several decades. In addition, significant disparities exist in several cancer risk behaviors, including obesity, intake of fruits and vegetables, leisure time physical activity and cancer screening. Addressing these disparities require successful development of relationships with minority communities to partner in the research process, in order to understand areas of critical need and develop interventions that are compatible with this community. In this manuscript we describe Project CHURCH (Creating a Higher Understanding of Cancer Research and Community Health), a collaborative partnership between The University of Texas MD Anderson Cancer Center and Houston-area African American churches. Project CHURCH was developed to understand disparities in cancer prevention risk factors and engage African Americans as partners in the research process. Using community-based participatory research principles, we describe the development and infrastructure of the research partnership, as well as how the church community has been engaged in the development and implementation of a large African American cohort study (*N* = 2,338). Finally, the characteristics of the cohort are presented along with cohort success in addressing community need while having significant contribution to the scientific literature. Project CHURCH serves as a valuable resource for cancer prevention in the African American community.

## Introduction

African Americans in the United States bear a disproportionate burden of cancer. They have the highest mortality rate of any racial and ethnic group for all cancers combined and specifically from malignancies of the lung and bronchus, colon and rectum, female breast, prostate, and cervix (1). Further, approximately 1 in 2 African American men and 1 in 3 African American women will be diagnosed with cancer within their lifetime (1). Moreover, significant disparities exist in several cancer risk behaviors, including smoking cessation, intake of fruits and vegetables, leisure time physical activity and cancer screening, with African Americans often suffering disproportionately from the adverse consequences of these behaviors (1–3).

Design and conduct of research to understand the causes of cancer disparities among African Americans is critical, yet remains a complex issue. It is widely recognized that health disparities have multiple levels of influence, from biological/genetic pathways to social conditions, culture, and public policy (4). Context, in particular the social, economic, and physical environment in which individuals live, plays an important role in health outcomes and behaviors. Thus, it is important to collect longitudinal data on a wide variety of contextual variables, in addition to those related to health behavior, among minority populations (5). Collecting such data can be particularly challenging as this requires sustained engagement with the African American community for participant recruitment and retention. Barriers to minority participation in research have been documented at length and include low trust in academic research and researchers, perceived limited benefit of participation, misperception about research, difficult study logistics, and economic and time constraints faced by potential participants (6). Building trust will also be important for future Precision Medicine Initiatives as they seek to engage diverse communities in research.

Addressing the barriers related to participation in research requires successful development of relationships with minority communities to partner in the research process (7, 8). African American churches have been engaged in cancer research for several decades; the church setting serves as a central aspect of community among many African Americans (9–11) thus has been recognized as an integral component of delivering health promotion and disease prevention services (9, 12–14) Further, roughly half of African Americans report attending church weekly (15), resulting in congregations spanning the socioeconomic spectrum (16) which provides extensive reach in this population. Church congregations often have resources such as active health ministries (17), stable membership over long periods of time (9), and members with deep social ties (16). Church-based health promotion interventions have resulted in increased physical activity (9, 18–20), increased fruit and vegetable intake (19, 21–24), and increases in cancer screening behaviors (e.g., mammography, colorectal cancer screening) (25, 26). However, developing such fruitful partnerships with churches is a delicate process that requires investment in time and resources; development of trust; and thorough, systematic planning (9, 27).

We illustrate the process of developing research partnerships with faith-based communities through description of a successful church-based partnership, Creating a Higher Understanding of cancer Research and Community Health (Project CHURCH). Project CHURCH, developed in 2009, is an ongoing collaborative partnership between The University of Texas MD Anderson Cancer Center and Houston-area African American churches to understand disparities in cancer risk factors and to engage African Americans as partners in the research process in order to reduce barriers to research participation and improve research outcomes. The partnership framework involved five aims: (1) partnership development and church engagement; (2) cohort study implementation; (3) dissemination of Project CHURCH findings to church partners; (4) implementation of cancer control programs; (5) development and evaluation of cancer prevention interventions; and (6) provision of research experiences to minority trainees interested in cancer disparities. This information may benefit researchers who seek to create sustainable research partnerships with faith-based organizations in order to conduct research with minority populations.

## Materials and methods

### Aim 1: partnership development and church engagement

#### Framework for project CHURCH

Conducting research with faith communities necessitates a community-based participatory research approach (CBPR), as involving church partners is critical throughout the research process and for improving health (28). CBPR is an integration of eight distinct principles (28) including enhancing communication, building trust and capacity among all partners, recognizing the unique strengths of each partner, and equitably sharing resources. These principles guided development of the Project CHURCH partnership, with our specific strategies detailed so as to provide a strong example of applied approaches of CBPR methods.

Project CHURCH was initiated in December 2008 with an African American mega-church (Church A) with over 15,000 members at that time. MD Anderson approached the church with the idea of developing a partnership to address cancer health disparities among African Americans, a population that leads in most cancer risk indicators (e.g., mortality, low cancer screenings, obesity, etc.) in Houston (29, 30). Church A had a long history of prior collaborations (e.g., recruitment of members to clinical trials) with MD Anderson and therefore was an obvious choice to approach for partnership. In 2012, we included two additional African American churches (Churches B and C with ~ 5,000 members each) into the Project CHURCH family, all utilizing the same framework and processes.

#### Project CHURCH churches

Churches were located primarily in southwest Houston. We started the partnership with Church A and decided to partner with churches within a short drive to Church A, as Church A is the setting where all Project CHURCH participants were consented and enrolled in the cohort. Members lived across the Greater Houston metropolitan area, with the majority living in Brazoria, Montgomery, Galveston, Fort Bend, and Harris counties.

#### Partnership development

One of the most important initial steps in working within the African American community is building trust and establishing credibility (9). We spent 1 year building our partnership, discussing questions and concerns about motivations/intentions and deliverables, all designed to develop trust and transparency and to answer the ultimate question of whether our academic institution could be trusted to engage in this partnership in the true spirit of CBPR. As outlined by Campbell et al. (9), trust building activities included showing outward signs of caring and compassion, and engaging in two-way dialogue about community concerns and in particular, concerns about the need for improved relationship between the African American community and research organizations. When possible, research staff attended church worship services and programs, responded to church requests for cancer control programming (e.g., requests for physician-speakers for events), and navigated church members to community screening services. In this phase, the engagement goal was to establish a strong foundation upon which to build this partnership. At this juncture, we decided to partner to conduct a cohort study designed to understand disparities in cancer prevention risk factors. A cohort was chosen because it allowed us to: (1) understand disparities over time (i.e., instead of at just one point in time); (2) include the entire church congregation in the research due to the number of participants required; and (3) provide data on the health of the congregation to the church on an annual basis so they could design health promotion programs. We also decided that we would share research findings with the church larger community (those that participated in the cohort and those that did not), and navigate church members to cancer services including screening and treatment. Operationalization of these aims was achieved through the development of an advisory board.

#### Advisory board development

The design of Project CHURCH was highly collaborative. One of the most important strategies employed was the use of a community advisory board. Advisory boards, common in community based research, provide structure for community members to voice their concerns in research and have potential to provide valuable direction to community-focused study design (31). Our experience led us to convene a church and scientific advisory board with Church A to ensure that it would be in line with expectations of the church, be mutually beneficial, and enhance the relevance of the partnership to the wider church body. The purpose of this board was to oversee the development of all cohort study procedures, materials, and content, and guide the implementation of cancer control programs and church navigation to services. The advisory board was selected by the church pastor and was comprised of eight church members from various backgrounds (e.g., cancer survivor) and leadership roles (e.g., health ministry leader), and included three faculty members from MD Anderson's Department of Health Disparities Research. The advisory board met monthly in the first year of the study and on an as-needed basis thereafter.

The advisory board was chaired and facilitated by the study PI (Dr. McNeill). All of the board members participated in education and training in CBPR and research, including specialized readings and in-person workshops. We also discussed CBPR principles that would guide the development of the partnership and implementation of the cohort study, such as co-learning, balance between research and action, and knowledge dissemination (31, 32). During each meeting we discussed partnership successes and challenges and planned future programs/services. There was no formal decision-making process; we tried to reach consensus at all times. Church advisory board members were compensated for their time with an annual $200 honorarium (paid starting in the 2nd year). Separate Advisory Boards for churches B and C were formed and operated under similar principles.

## Results

### Aim 2: collaborative cohort study

Together, the advisory board developed study procedures for conducting a cohort study. The purpose of the cohort study was to examine intrapersonal, interpersonal, institutional, community, and public policy cancer risk factors that were thought to contribute to racial/ethnic disparities in cancer-related outcomes. Formal data collection was initiated in December 2008, and by July 2009, we enrolled 1501 African American participants from Church A to participate in the cohort. Churches B and C joined the Project CHURCH cohort study in 2013 resulting in a final cohort size of *n* = 2,338. The Project CHURCH cohort participants are not representative of their respective churches or African Americans in Texas as this was not its purpose. Rather, the cohort was a means to engage church members in research, identify salient cancer risk factors among African American church members in Houston, and develop interventions based on those findings.

#### Recruitment strategy and data collection

We developed a comprehensive recruitment strategy. Study information was shared via posted flyers at the church, on the church website, in the church newsletter and in the video announcements aired during church services. Advisory board members also networked with their respective within-church organizations (e.g., choir, usher ministry, health ministry), sharing details about the study and inviting members to participate. To kick-off study recruitment, each pastor introduced the PI to the congregation and the purpose of the partnership. Each pastor allowed her to address the congregation from the pulpit, asking for their participation. We hosted a health fair on the study kick-off day; church members had access to preventive health screenings, health information, and healthy food and physical activity demonstrations. Advisory board members and members of the research team enrolled participants on that day or invited participants to sign up for the study. At Church A we received over 500 sign-up forms on kick-off day; Churches B and C had over 190 and 300 sign-up forms, respectively. When necessary, we actively recruited participants after church services and other weekday events (e.g., bible study).We are unable to provide enrollment rates as those data were not collected.

In order to embed our work in the community, our initial CHURCH partner (Church A) allowed Project CHURCH to have permanent space in the church, which we called our Project CHURCH office. This would reduce barriers to participation and allow Project CHURCH to meet the needs of the participants in a familiar setting, rather than having to travel to the medical center. All participants, across all three churches, were consented and enrolled at the Project CHURCH office. In addition, all research staff was African American and received training in cultural competence with faith-based organizations. Our goal was to provide excellent customer service in the enrollment process by being knowledgeable about the study protocol, following up on questions or concerns raised during enrollment, and projecting friendliness to create a positive research experience for the participant.

To participate in the cohort study, participants had to be at least 18 years old, have a working telephone number and an address where they could be reached, and attend the church. These broad criteria were used to enhance participation and inclusiveness and limit the number of people who could be excluded—again to enhance participation. Participants did not have to be members of the church but needed to have attended the church at some point. Participation by families was allowed and even encouraged. As African Americans have a strong family history of cancer, this was an area we wanted to investigate. Thus, our broad inclusion criteria also removed the need to have the church share their membership roster, some of which were hesitant to do so at the beginning of the relationship. Participants were aware that participation entailed an annual survey for up to 5 years (pending funding availability).

#### Study procedures

Participants were scheduled for appointments at the Project CHURCH office. After consenting and enrolling, participants' height and weight were measured. Participants then completed a computer-assisted survey. Upon completion of the survey, participants were offered brief health education, could visit the Project CHURCH cancer prevention library (kiosk with health information) and were then compensated with a $30 Visa debit card and received small incentive items.

#### Study questionnaire

The goal of the cohort was to better understand the complex interaction between individual, social and environmental determinants of cancer risk in African Americans. We wanted to go beyond traditional approaches that examine individual level risk factors (e.g., behaviors) to potentially understand how context, such as neighborhoods in which African Americans live and the mental health risks of stress and depression interact with cultural, biological, and behavioral processes at play. We adapted the cancer prevention conceptual model developed by Sorensen and Emmons (5) to include individual factors (e.g., financial strain, perceived stress, perceived racism, residential segregation), interpersonal factors (e.g., social support, social networks, social standing ladder), organizational factors (e.g., church membership, job strain, health care access), and neighborhood/community factors (e.g., neighborhood safety), in addition to lifestyle cancer risk factors (e.g., diet, physical activity, tobacco use), health conditions (e.g., obesity, diabetes, hypertension), and cancer screening. We included brief, validated questionnaires where possible; however, this was not always possible given our coverage of topics and direction from the advisory board to keep the survey completion time to 45 min to 1 h to minimize respondent burden. For administration, we used a computer-based assisted survey with a computer read aloud (with headphones) option for those with limited reading proficiency or who needed audio help to complete the survey

The content of the questionnaire was negotiated with the advisory board yearly, introducing new constructs (e.g., loneliness) and removing and retaining old ones (e.g., cancer risk behaviors). Each church questionnaire included common questions noted above as well as items of interest specific to their congregation. For example, one church had a significant number of cancer survivors and wanted to include an extended questionnaire on survivorship and another church was interested in experiences of pain among the participants.

#### Biological specimens

Beginning in the 2nd year of the study, after substantial trust had been built with the church, we asked participants to provide a saliva buccal sample to be banked and used for future cancer research. The advisory board had many concerns about the safe collection and use of this data, but approved the collection as they wanted to ensure that their research participation was meaningful and that these data would help contribute toward major advancements for African Americans in cancer research. The buccal sample was included as an optional procedure in Church A only; over 91% of participants provided a sample in Year 2. All participants provided informed consent for the biobanking. Trained research staff read through the informed consent document and answered any questions participants may have had. Per the consent document, participants at any time can withdraw their study participation and their samples from the protocol. Only IRB-approved ancillary studies will be approved for use of the samples and the safety and monitoring of the samples will be overseen by the IRB. The samples are genotyped, stored at the MD Anderson Center for Translational and Public Health Genomics, and available for ancillary study use.

#### Follow up

Our accrual goal for Church A was 1,000 participants, which we adjusted to 1,500 given the positive response. We were not prepared for such eager participation and had to quickly hire additional staff to meet recruitment demands. We completed enrollment in 6 months. We did not have explicit goals for Churches B (*N* = 410) and C (*N* = 427); however, we enrolled participants in each church for a 4 month period. We completed 3 waves of data collection with Church A, achieving a 93% retention rate from Year 1 (baseline) to Year 2 and an 82% retention rate through Year 3 (33). We attribute these rates to the various methods of community engagement (e.g., advisory board, kick-off protocol) and retention strategies (e.g., Project CHURCH office, regular communication via newsletter and feedback, compensation) employed. Data collection for Church A included annual surveys, anthropometric measures, buccal saliva sample (Year 2/Church A), and accelerometer data (Year 2/Church A; targeted *N* = 500). Churches B and C completed data collection at one time point (Year 1/survey and anthropometric measures) due to limited funding.

#### Overview of baseline (year 1) findings

As of December 31, 2013, *N* = 2,338 participants have enrolled in the Project CHURCH cohort study (data is presented for 2,254 due to missing data). Selected Year 1 characteristics, described by sex, are presented in Tables 1–4. For all churches combined (Table 1), 70% of participants were female, reflecting the general composition of African American church goers. The average age was 49.1 years (±16.3) for males and 52.3 (±13.8) for females. The cohort was highly educated, with 45% of participants having a bachelor's degree or higher. About 30% had an annual household income of $40,000 or less. Regarding cancer risk factors, the majority of participants were never smokers (76.3%) and 7.4% were current smokers, with more men than women reporting current smoking (11.9% vs. 5.8%). The prevalence of obesity was significant; 56.6% were obese, defined as a body mass index of 30 kg/m^2^ or greater, with a greater proportion of females than males being obese (59.4% vs. 48.6%). On average the cohort population did not meet fruit and vegetable recommendations (14.9%). Participants reported low perceived stress, high experience with discrimination (cut point ≥ 19), and 17.1% reported depressive symptoms. These findings varied slightly by church.

**Table 1:** Project CHURCH participant characteristics (All Churches, *n* = 2,254).

**Characteristics**	**Mean (SD)/Percent**		
	**Male**	**Female**	**Total**
Sex	26.1	73.9	100.0
Age	44.6 (14.5)	46.3 (13.4)	45.8 (13.7)
**EDUCATION**			
≤ High school	22.8	13.5	15.9
Some college	39.0	39.6	39.5
≥ Bachelors degree	38.2	46.9	44.6
**ANNUAL HOUSEHOLD INCOME**			
<$40,000	25.3	32.2	30.4
$40,000–$79,999	32.4	37.5	36.2
≥$80,000	42.3	30.3	33.4
**SMOKING**			
Current Smokers	16.6	7.4	9.8
Former smokers	17.5	13.8	14.8
Never smokers	65.9	78.8	75.5
**BODY MASS INDEX**			
Normal	15.5	17.7	17.1
Overweight, not obese	36.6	26.2	28.9
Obese	47.9	56.1	54.0
**SELF-REPORTED PA LEVEL**			
Low	18.6	29.2	26.5
Moderate	20.5	33.0	29.9
High	60.9	37.8	43.6
**SERVINGS OF FRUITS AND VEGETABLES/DAY**			
<5 servings per day	86.1	82.9	83.7
≥5 servings per day	13.9	17.1	16.3
Servings of red meat/week	5.9 (4.5)	4.8 (4.1)	5.1 (4.2)
Perceived stress	4.6 (3.0)	4.8 (3.1)	4.8 (3.0)
Discrimination	21.1 (8.2)	19.8 (7.0)	20.2 (7.3)
**DEPRESSIVE SYMPTOMS**			
No	83.7	78.3	79.7
Yes	16.3	21.7	20.3

*Physical activity = International Physical Activity Questionnaire (IPAQ)*.

*Perceived Stress = Perceived Stress Scale*.

*Depressive Symptoms = Center for Epidemiological Studies Depression 10-item scale*.

*Discrimination = Day-to-Day Unfair Treatment Scale*.

**Table 2:** Project CHURCH participant characteristics (Church A, *n* = 1,467).

**Characteristics**	**Mean (SD)/Percent**		
	**Male**	**Female**	**Total**
**Age**	44.3 (13.7)	45.5 (12.5)	45.2 (12.9)
**EDUCATION**			
≤ High school	15.6	11.2	12.3
Some college	42.0	38.3	39.2
≥ Bachelors degree	42.3	50.5	48.4
**ANNUAL HOUSEHOLD INCOME**			
<$40,000	20.0	27.1	25.3
$40,000–$79,999	36.1	40.5	39.4
≥$80,000	43.9	32.4	35.3
**SMOKING**			
Current Smokers	15.4	6.7	8.9
Former smokers	18.7	14.0	15.2
Never smokers	65.9	79.3	75.9
**BODY MASS INDEX**			
Normal	14.0	18.2	17.1
Overweight, not obese	36.8	27.4	29.8
Obese	49.2	54.4	53.1
**PA LEVEL**			
Low	15.7	31.7	27.7
Moderate	22.4	31.4	29.2
High	61.9	36.9	43.1
**SERVINGS OF FRUITS AND VEGETABLES/DAY**			
<5 servings per day	86.6	82.6	83.6
≥5 servings per day	13.4	17.4	16.4
Servings of red meat/week	5.8 (4.2)	4.8 (4.1)	5.1 (4.2)
Perceived stress	4.4 (3.0)	4.7 (3.1)	4.6 (3.0)
Discrimination	21.6 (8.3)	20.1 (7.0)	20.5 (7.4)
**DEPRESSION**			
No	84.6	80.0	81.2
Yes	15.4	20.0	18.8

*Physical activity = International Physical Activity Questionnaire (IPAQ)*.

*Perceived Stress = Perceived Stress Scale*.

*Depressive Symptoms = Center for Epidemiological Studies Depression 10-item scale*.

*Discrimination = Day-to-Day Unfair Treatment Scale*.

**Table 3:** Project CHURCH participant characteristics (Church B, *n* = 370).

**Characteristics**	**Mean (SD)/Percent**		
	**Male**	**Female**	**Total**
**Age**	40.7 (13.7)	42.6 (14.3)	42.1 (14.2)
**EDUCATION**			
≤ High school	48.6	26.2	32.7
Some college	28.0	41.8	37.8
≥ Bachelors degree	23.4	31.9	29.5
**ANNUAL HOUSEHOLD INCOME**			
<$40,000	51.5	51.7	51.7
$40,000–$79,999	24.3	30.9	29.0
≥$80,000	24.3	17.4	19.3
**SMOKING**			
Current Smokers	25.5	12.2	16.0
Former smokers	12.3	10.6	11.1
Never smokers	62.3	77.2	72.9
**BODY MASS INDEX**			
Normal	21.7	17.8	18.9
Overweight, not obese	35.8	22.8	26.6
Obese	42.5	59.5	54.5
**PA LEVEL**			
Low	21.8	22.5	22.4
Moderate	16.1	35.6	30.6
High	62.1	41.9	47.1
**SERVINGS OF FRUITS AND VEGETABLES/DAY**			
<5 servings per day	83.2	82.1	82.4
≥ 5 servings per day	16.8	17.9	17.6
Servings of red meat/week	6.2 (5.2)	5.2 (4.6)	5.5 (4.8)
Perceived stress	5.8 (2.7)	5.6 (3.0)	5.6 (3.0)
Discrimination	20.6 (8.1)	19.5 (6.5)	19.8 (7.0)
**DEPRESSION**			
No	77.0	67.6	70.2
Yes	23.0	32.4	29.8

Physical activity = International Physical Activity Questionnaire (IPAQ)

Perceived Stress = Perceived Stress Scale

Depressive Symptoms = Center for Epidemiological Studies Depression 10-item scale

*Discrimination = Day-to-Day Unfair Treatment Scale*.

**Table 4:** Project CHURCH participant characteristics (Church C, *n* = 417).

**Characteristics**	**Mean (SD)/Percent**		
	**Male**	**Female**	**Total**
**Age**	49.1 (16.3)	52.3 (13.8)	51.4 (14.6)
**EDUCATION**			
≤ High school	22.0	10.7	13.7
Some college	39.4	42.5	41.7
≥ Bachelors degree	38.5	46.8	44.6
**ANNUAL HOUSEHOLD INCOME**			
<$40,000	17.0	33.4	29.2
$40,000–$79,999	28.0	32.4	31.3
≥$80,000	55.0	34.1	39.5
**SMOKING**			
Current Smokers	11.9	5.8	7.4
Former smokers	18.3	15.6	16.3
Never smokers	69.7	78.6	76.3
**BODY MASS INDEX**			
Normal	14.7	15.6	15.3
Overweight, not obese	36.7	25.0	28.1
Obese	48.6	59.4	56.6
**PA LEVEL**			
Low	25.5	26.2	26.0
Moderate	17.9	36.4	31.6
High	56.6	37.4	42.4
**SERVINGS OF FRUITS AND VEGETABLES/DAY**			
<5 servings per day	87.2	84.4	85.1
≥ 5 servings per day	12.8	15.6	14.9
Servings of red meat/week	5.7 (4.6)	4.3 (3.7)	4.6 (4.0)
Perceived stress	4.1 (2.8)	4.8 (3.1)	4.6 (3.0)
Discrimination	19.7 (8.1)	19.2 (7.0)	19.3 (7.3)
**DEPRESSION**			
No	86.9	81.4	82.9
Yes	13.1	18.6	17.1

*Physical activity = International Physical Activity Questionnaire (IPAQ)*.

*Perceived Stress = Perceived Stress Scale*.

*Depressive Symptoms = Center for Epidemiological Studies Depression 10-item scale*.

*Discrimination = Day-to-Day Unfair Treatment Scale*.

### Aim 3: dissemination of project CHURCH findings with church community

Communities are potentially vulnerable in community-academic partnerships. There are real possibilities for community organizations to invest time and resources in scientific research projects and not receive anything beyond the short-term benefit of the research (e.g., health promotion intervention). Ongoing communication and sharing of research results with the community is an ethical responsibility of researchers and critical to relationship maintenance (32, 34), however these efforts often suffer due to time and resource constraints. CBPR projects frequently have long timelines, making dissemination difficult as resources to support manuscripts and community-focused dissemination may not be available at the conclusion of the funding period (34). The Project CHURCH team employed strategies that would ensure information would reach our population.

#### Newsletters

Project CHURCH participants received brief health education during survey data collection and four mailed cancer prevention newsletters specifically designed for the study. Newsletters included a note from the PI, information/guidelines on a cancer prevention topic (e.g., screening), a healthy recipe, and a feature article highlighting an advisory board member and their support for Project CHURCH.

#### Annual report

We provided an annual report to each church and all cohort participants (mailed) which presented aggregated findings, included the most up-to-date cancer prevention recommendations, and outlined future directions. For broad dissemination, this report would be included on the churches' websites. Report content was determined with the pastor and advisory board after preliminary findings were shared and discussed.

#### Individual feedback

Based on guidance from the advisory board in 2009, Project CHURCH participants also received individual feedback on select cancer prevention behaviors based on their survey responses. We shared information regarding whether they met cancer recommendations (e.g., engaging in 150 min of physical activity) and, if necessary, provided recommendations for how to meet them. Cancer prevention behaviors included physical activity, Body Mass Index, fruit and vegetable consumption, cigarette smoking, and colorectal cancer screening. This feedback was provided within 2 months of survey completion.

#### Published research

Since 2011, the Project CHURCH cohort has published 25 papers with an additional 8 under review. We have made significant contributions to understanding the role of genetic variants as well as that of individual, social and environmental factors on cancer risk (35–41). For example, in one study we found a positive relationship between the density of fast-food restaurants and body mass index for lower income study participants, indicating the influence of neighborhood environment on disease (36). In another study we found that financial strain, or perception of income inadequacy, was positively associated with specific cancer risk factors (insufficient physical activity and smoking) as well as the total number of cancer risk factor behaviors (42). Over the next several years we plan to continue investigation of major research questions regarding African American cancer risk; including health service utilization, genetic predispositions, energy balance (diet, physical activity, obesity), and tobacco use and their associations with individual (e.g., perceived stress, depression), social, and environmental factors (e.g., neighborhoods, perceived discrimination).

### Aim 4: implementation of cancer control programs and services

It is important to note and understand the rationale or purpose for communities to participate in research. Given that one of the strongest motivations for participation is how the research may impact participants' own lives or that of the community (43), considerable attention must be paid to personal and/or community benefit. Project CHURCH pastors and advisory boards emphasized the need to provide immediate direct and tangible benefits for participants. This is in line with CBPR tenets, which recommend that services be provided as part of or ancillary to a research project (44). We developed tactics to deliver services in high-need areas identified by the advisory board, church, and research findings.

#### Patient navigation

In response to community requests for timely cancer risk and prevention information, we provided patient navigation services to all CHURCH participants as well as members of each congregation. We helped break down the barriers to obtaining prevention services and getting cancer care. Church members could call the Project CHURCH office and be automatically connected with our Project CHURCH patient navigator. We have provided patient navigation services to almost 100 individuals, with concerns ranging from how to get mammograms in the community and learning more about cancer care at MD Anderson to visiting participants/church members receiving care at MD Anderson.

#### Church programming

MD Anderson and Project CHURCH churches jointly implemented at least 2–3 cancer prevention programs each year. Program activities included an 8 week smoking cessation course (with free nicotine replacement therapy), an 8-week salsa class, a mental health workshop, and cancer expert speakers' series. We built on the strengths of the church, primarily partnering with and supporting existing health, youth, women's and men's ministries with ongoing or new health-related programs.

### Aim 5: development and evaluation of cancer prevention interventions

To achieve balance between research and action, we developed and implemented cancer prevention interventions throughout the partnership. While traditional cohort studies desire to maintain the purity of the cohort and not interfere by providing interventions, our cohort approach explicitly sought to implement research programs that would address relevant health problems. Using our findings we have designed and tested innovative interventions, an example being the Healthy Habits Study, a randomized trial to improve diet and physical activity in overweight African American adults (funded by a local foundation). This study resulted from preliminary analyses of the cohort data indicating high prevalence of overweight and obesity and low fruit and vegetable intake, potentially increasing cohort risk for cancer. Through the Project CHURCH database, we were able to enroll over 200 participants in less than a year.

Project CHURCH has established an Ancillary Studies Committee (ASC), composed of MD Anderson faculty and an advisory board representative, which evaluates new proposed research. All research projects, including manuscripts, grants, or protocols, must be relevant to African American health locally or nationally and have potential church benefits. Through our ASC, we were able to provide biospecimens (buccal samples) for use as controls in an African American lung cancer Genome-wide association study (GWAS) at the National Cancer Institute (38, 39, 45). Project CHURCH also provides recruitment support to investigators who are interested in conducting research with African Americans in Houston who are at MD Anderson and other Texas Medical Center institutions (e.g., University of Houston, Rice University). Project CHURCH participants all agree to be contacted for future research projects. To date, we have assisted at least 7 faculty, which has resulted in almost 300 African Americans enrolling in cancer prevention research.

### Aim 6: provide research experiences to minority trainees interested in cancer disparities

It is well documented that there is a limited number of well-trained racial/ethnic minority researchers engaged in cancer research (46). Increasing these numbers is paramount in order to address current and future health disparities. CBPR programs should promote co-learning that facilitates transfer of knowledge, skills and capacity. To address this need, in 2011 we initiated a program to identify student trainees attending Project CHURCH churches for research experience opportunities at MD Anderson. Students from varied disciplines (e.g., Nursing, Public Health, Psychology), educational levels (high school through postdoctoral fellows), and universities (e.g., Lamar University, Sam Houston State, Texas Southern University, Prairie View A&M University, University of Houston, Rice University, and UT Health Sciences Center School of Public Health) gain experience in CBPR, cancer prevention, and health disparities research. Trainees engage in applied, hands-on research experiences (i.e., participant recruitment and enrollment, data collection, intervention implementation, as well as data management, analysis and interpretation) that develop and deepen their understanding of cancer disparities among African Americans and ultimately enhance understanding of how classroom concepts are operationalized. Students participate throughout the year, including 10-week summer fellowships and academic year placements. In the past 4 years, over 40 students have received research experiences with Project CHURCH. Our trainees have obtained tenure-track faculty positions, enrolled in master's and doctorate public health and psychology programs, and have pursued careers in cancer prevention research and/or practice. Six papers have been published in peer-reviewed journals with students as the first author utilizing the Project CHURCH cohort data. This novel training program addresses the problem of workforce diversity by training a cadre of talented racial/ethnic minorities to pursue careers in cancer disparities research. Based on this success, we have recently applied for a formal training grant to expand the training program and provide enhanced training experiences for students.

## Discussion

The goal of this partnership was to enhance engagement of African Americans and faith-based organizations in cancer research. Toward that end, we have created a unique partnership that could be a model for other cancer researchers interested in gaining substantial participation and active support of African Americans. Given the large number of African Americans that report church attendance, engaging in research partnerships with faith organizations is important to expand reach and ensure the relevance of cancer research in this population. The engagement principles and activities, research methodology, intervention development, and student training aspects of this partnership aided us in overcoming and addressing known barriers to participation of African Americans in cancer research.

Our work builds upon the foundation of similar partnerships with African Americans such as the Jackson Heart Study (cardiovascular disease) and Southern Community Cohort Study (cancer risk) (47–49). To our knowledge, our research partnership is the first to collaborate with African American churches as part of its methodology, to utilize CBPR as a guiding framework, and to provide interventions to address data-driven and community-expressed health concerns. Our community-centered approach and CBPR foundation is replicable and can be achieved in other populations and geographic locations to develop a comprehensive agenda to address cancer disparities. It begins with a strong desire to have honest and thoughtful communication that addresses mistrust of the research process. It also requires people willing to spend the time, energy and effort to plan and implement research that benefits the community (9). These were our initial tools for Project CHURCH. We have met all our partnership aims and continue to implement them as we grow the partnership, contribute new knowledge about African American cancer risk, and develop relevant interventions.

As noted, our Project CHURCH partnership was developed over many years, starting with Church A. We were able to streamline the process with Churches B and C due to experience gained in conducting this type of research with Church A. We have learned that engagement is not just with individuals for the cohort study (who can move or be lost-to-follow up), but rather in the partnership with the church which is the vehicle that will promote ongoing interest in current and future research and activities. Other churches, aware of our success, are now eager to participate in Project CHURCH. We have recently been contacted by a church with a large membership that recognizes the value of such a partnership to their church and immediate community. Through partnership, research, and outreach activities, we are potentially “connecting” with over 20,000 African Americans in Houston and the surrounding church neighborhoods.

Church members and participants have shared several reasons for their participation and engagement in Project CHURCH activities. They liked the notion of the partnership with their church, adding a level of trust and credibility that we might not have gotten if we conducted this solely as an academic institution. Many expressed altruistic reasons for participating; citing a family history of cancer and the need to help fight this battle, and service to God and mankind. Participant reactions included, “You guys are helping us help ourselves” and “We [my wife and I] will help even if it is for nothing. I wish I could do more. I am always grateful.” They also noted the great customer service during recruitment and enrollment and at all Project CHURCH activities. We have created a strong foundation that will be vital moving forward in assessing additional/multiple cancer risk factors and developing innovative, impactful cancer prevention intervention for African Americans. We have advisory boards at each church that are engaged and committed, met accrual goals with ease, and continue to identify interventions and programmatic activities to address survey findings and church concerns. Project CHURCH churches and cohort members have demonstrated a strong commitment to improving their own health as well as that of their communities.

An endeavor like this is not without limitations. While we utilize a CBPR foundation for our partnership, we sometimes struggle with consistent adherence. Project CHURCH is an ambitious undertaking by all parties. Churches' focus on health is in line with their mission to bring people closer to God, however research, itself, is not. Thoughtful decisions are therefore needed regarding the type of research in which to engage. For example, we recently implemented a mind-body, yoga-type intervention to better understand its use/feasibility for African Americans—who report lower participation in these types of activities (50). A culturally-adapted intervention was implemented after lengthy discussion of methods to provide this type of intervention to church members while honoring religious sanctity (given the incompatibility between yoga, with foundations in Hindu, and Christianity). Respect for and sensitivity to religious differences is a central tenet of working with African American churches (9). Although the majority of the research staff was Christian, in every church the structure, norms and culture are different. As we continue to learn about the culture of African American churches, researchers must recognize that not everything we want to examine or explore will be accepted or feasible. Likewise, our church partners are gaining knowledge and experience in cancer research, sometimes facing challenging notions of harm vs. benefit, such as the use of biobanking protocols and protections for church members who participate. We are also not yet addressing the root causes of health inequities: poverty and social injustice. We hope that through long-term engagement with additional community based organizations, academic centers, and local city and state leaders, we can bring attention to these root causes and identify strategies to address them. Finally, our cohort data is not representative of African Americans in general. Although almost 50% of African Americans report weekly church attendance, with another 36% that report attending once or twice a month/a few times per year (15), cohort data may not generalize to African Americans in Texas or in the US. However, this is the first cohort of this nature in Texas, has served as a means for community engagement, and is an important way to engage many African Americans. As our main focus was to describe how to effectively engage large populations of African American church-goers, it is equally important to develop effective strategies to engage racial/ethnic minorities and other populations without strong church ties. The settings, engagement strategies used and research that seeks to be conducted will all need to align.

This approach also hold promise for Precision Medicine initiatives. The NIH Community Engagement Team of the Precision Medicine Initiative noted at their July 2015 meeting that precision medicine may be uniquely critical to reducing health disparities and inequities in health outcomes. The PMI cohort can help “identify and test strategies to overcome study participation barriers” and “examine neighborhood factors that contribute to health disparities.” However, none of this possible without adequate participation of these diverse communities. This paper describes a method for going to where the people are, i.e., “boots on the ground” method of engagement that works, with high recruitment and retention rates and impactful efforts to make the this research relevant to their needs and concerns.

Planning for sustainability has been central to Project CHURCH as it enters its 10th year. Ongoing research partnerships require continued funding and plans for such were discussed among the research team and advisory board before initiation of the cohort study, as recommended by others (31, 32). Project CHURCH began with initial funding by MD Anderson and philanthropy from the Department of Health Disparities Research. We have actively pursued approaches to maintain a long-standing partnership-incorporating capacity building, initiating pilot studies, and applying for funding opportunities that serve to benefit both institutions and expand the network of participating churches in the cohort. These grants maintain commitment to CBPR principles, requiring that churches receive financial subcontracts and incorporate training and capacity building in research. To date we have received additional philanthropy from foundations and individuals, peer-reviewed grant funding from NIH, and an administrative supplement to our Cancer Center Support Grant. Our future plans are to enhance and expand the partnership to include additional churches in the Houston area through a recently-funded NIH conference series grant (1R13HD080934-01) to build a coalition of churches to address cancer disparities.

Project CHURCH data are available for research. To request data, biological samples or to join the partnership for collaborative research, please email Project CHURCH at church@mdanderson.org.

## Ethics statement

The protocol was approved by the Institutional Review Board at MD Anderson Cancer Center. All subjects gave written informed consent in accordance with the Declaration of Helsinki.

## Author contributions

LM, LR, JV, LS, and DW contributed conception and design of the study. CR organized the database. NN performed the statistical analysis. LM and KE wrote the first draft of the manuscript. All authors contributed to manuscript revision, read and approved the submitted version.

### Conflict of interest statement

The authors declare that the research was conducted in the absence of any commercial or financial relationships that could be construed as a potential conflict of interest.
